# A227 CTLA-4 DEFICIENCY PRESENTING AS CROHN DISEASE IN A TEENAGE BOY WITH MULTI-SYSTEM INVOLVEMENT : A CASE REPORT

**DOI:** 10.1093/jcag/gwac036.227

**Published:** 2023-03-07

**Authors:** H M R Binomar, C Biggs, C Yang, A Amid, D Schraeder, O Guttman

**Affiliations:** 1 Pediatrics- Gastroenterology; 2 Pediatrics - Allergy & Immunology; 3 Pediatrics- Respirology; 4 Pediatrics- Oncology; 5 Neurology , British Columbia Children's Hospital , Vancouver , Canada

## Abstract

**Background:**

Cytotoxic-T-lymphocyte-antigen-4 is a negative immune regulator. CTLA-4 deficiency can cause a complex immune dysregulation syndrome that may present as inflammatory bowel disease.

**Purpose:**

**Case Description:**

A 14-year-old male presented with several years of abdominal pain, diarrhea, short stature and poor weight gain. Endocolonoscopy diagnosed Crohn disease, with patchy chronic colitis, an ileal granuloma and active duodenitis with intraepithelial lymphocytosis and villous blunting. MRE showed multiple abnormal small bowel loops. Past history included appendicitis, immune thrombocytopenic purpura and an episode each of *Aeromonas*, *Y enterocolitica* and *C difficile* colitis. Family history was negative for consanguinity, autoimmune conditions or immune defects.

The patient received sulfasalazine, exclusive enteral nutrition and then budesonide MMX with limited response. His pre-biologic workup identified bilateral pulmonary nodules, which were negative for infection or malignancy on further investigation. He began Adalimumab, and after 2 months had nearly normalized his fecal calprotectin to 90 ug/g. His history, together with chronic neutropenia and hypogammaglobulinemia (IgG 3.3 g/L, IgA 0.29g/L) led to a comprehensive immune and cytopenia panel, which revealed heterozygous pathogenic variants in the CTLA4 gene c.424G>C, p.(Gly142Arg), associated with CTLA-4 deficiency related immunodeficiency. Parental testing was negative, indicating a *de novo* mutation.

The patient was subsequently admitted to hospital with severe headache and transient aphasia. Brain MRI found hyperintense foci concerning for CTLA-4 deficiency-associated CNS inflammation. He had papilledema and lumbar puncture found increased ICP. During this admission he developed *Salmonella* sepsis. Repeat chest CT found extensive worsening multifocal parenchymal lesions. Extensive investigations to exclude malignancy or infection were negative, and the lung findings were felt to be Granulomatous and Lymphocytic Interstitial Lung Disease. He had elevated soluble IL-2 receptor level (2884 U/ml) and elevated CD4+PD1+ T cells in peripheral blood. Adalimumab was stopped, and he received IVIg, then Diamox with resolution of headache, followed by pulse methylprednisolone. He has since been maintained on Abatacept (a soluble CTLA-4 analog that inhibits T-cell activation). Follow up soluble IL2 receptor level was normal. PJP prophylaxis was started, as well as prophylactic dosing of IVIg and regular screening for EBV and CMV.

**Method:**

-

**Result(s):**

-

**Conclusion(s):**

**Discussion:**

Our patient had CTLA-4 deficiency and immune dysregulation affecting multiple organs (CNS, lung, and intestinal inflammation, autoimmune neutropenia and previous ITP). Abatacept is the preferred steroid-sparing treatment of enteropathy in CTLA-4 deficiency. Soluble IL2 receptor level is an indicator of T cell activation used for treatment monitoring. This case illustrates the importance of considering immune defects even in older children presenting with apparent IBD.

**Please acknowledge all funding agencies by checking the applicable boxes below:**

None

**Disclosure of Interest:**

None Declared

INTESTINAL DISORDERS

